# Reinforcement Learning for Cathode Material Design Through Sequential Decision-Making Frameworks

**DOI:** 10.3390/nano16160981

**Published:** 2026-08-10

**Authors:** Taimoor Muzaffar Gondal, Muhammad Qasim, Yasir Arafat

**Affiliations:** 1 School of Engineering, Edith Cowan University, Joondalup, WA 6027, Australia; t.gondal@ecu.edu.au; 2 Faculty of Science, Bahauddin Zakariya University, Multan 60000, Pakistan; qali645@gmail.com

**Keywords:** reinforcement learning, cathode material design, lithium-ion batteries, sodium-ion batteries, inverse materials design, reward design, autonomous experimentation, closed-loop discovery

## Abstract

The cathode material design is a persistent challenge in the development of next-generation rechargeable batteries. The cathode performance is critically influenced by certain key parameters, i.e., composition, crystal structures, ion transport, and degradation behaviour. Moreover, techno-economic and sustainable considerations also play a pivotal role in the viable cathode material design. In recent years, the integration of static machine learning models with conventional experimental techniques has significantly enhanced the cathode material design. However, the sequential nature of cathode discovery has not been fully captured by these techniques as they do not update their decision strategy based on prior outcomes. In this review, reinforcement learning (RL) as a decision making technique for cathode material design has been evaluated. Firstly, cathode design space, including major cathode families, optimisation objectives, and key material variables have been explored. Afterwards, cathode discovery has been presented in terms of RL states, actions, rewards, policies, environments, constraints, and feedback. The key focus of this review is to analyse how RL can support the composition selection, dopant, and crystal structure optimisation. The review also discusses the current limitations of RL based cathode design including data scarcity, dataset bias, limited cathode specific benchmarks, reward function design, physical validity, and experimental validations. The future directions have been proposed for physics informed and experimentally validated RL infrastructure that integrates the density functional theory, molecular dynamics, artificial intelligence and human expertise.

## 1. Introduction

In rechargeable batteries, cathode materials are among the most critical components that directly influence electrochemical performance, cost, safety, and scalability. In lithium-ion batteries (LIBs), the cathode largely determines the operating voltage, reversible capacity, energy density, thermal stability, and long-term degradation behaviour of the full cell [1]. Major cathode families, i.e., layered transition metal oxides, polyanionic compounds, and sodium-ion cathodes etc., offer different trade-offs between capacity, voltage, cycle life, safety, and raw-material sustainability [2]. For example, Ni-rich layered oxides such as NMC and NCA are attractive for high-energy applications, but they are limited by structural instability and electrolyte reactivity at high states of charge [3]. In contrast, olivine and polyanionic cathodes generally offer improved safety and structural robustness, but often suffer from lower electronic conductivity or lower energy density compared with layered oxides [1]. The design of improved cathode materials is a comprehensive multi objective problem. A candidate cathode must satisfy electrochemical, thermodynamic, kinetic, mechanical, interfacial, economic, and environmental criteria. These requirements are strongly coupled with each other so optimising one parameter frequently compromises another. For instance, increasing Ni content may enhance capacity but tends to worsen structural and surface stability. Doping may suppress detrimental phase transitions but can reduce practical capacity. Similarly, surface coating may improve interfacial stability but risks introducing transport resistance. Notably, temperature and atmosphere directly influence the cation ordering, phase purity, and electrochemical performance. Cathode discovery consequently involves a high-dimensional design space. Key material and processing variables interact in complex and often unpredictable ways. Conventional trial-and-error experimentation is therefore insufficient to navigate this space systematically. The challenge is further compounded by the rapid expansion of cathode chemistries, from Co-reduced layered oxides to high-entropy and Na-ion systems. As a result, the number of viable candidate materials has grown far beyond what conventional experimental approaches can realistically screen.

Computational screening, density functional theory (DFT), molecular dynamics, high-throughput calculations, and machine learning (ML) have increasingly been used to accelerate battery-material discovery. In battery informatics, ML models are commonly used as surrogate functions to approximate expensive calculations or experiments. It enables faster prediction of voltage, stability, diffusion, capacity, lifetime, and degradation-related properties [4,5]. For cathode materials, data-driven approaches have been applied to property prediction, composition optimisation, phase screening, and voltage estimation etc. Liow et al. demonstrated a machine-learning-assisted synthesis workflow for LIB cathode materials, where expert-derived descriptors and inverse design were used to establish experimental parameter-property relationships and guide the fabrication of high-energy-density cathodes [6]. Similarly, voltage mining studies have shown how calculated cathode voltage data and ML models can be combined to identify chemical trends and predict voltage from charged and discharged compositions [7]. These developments show that ML can reduce the cost of screening and accelerate the interpretation of structure, property, and performance relationships. However, most supervised ML approaches remain fundamentally static. They generally learn a mapping from descriptors to target properties using existing data, after which the trained model predicts the performance of new candidates. This is useful for screening, ranking, and interpolation within known design spaces, but it does not automatically define a sequential design strategy. In cathode discovery, the next useful decision is rarely just, which candidate has the highest predicted capacity? Rather, it depends on a several factors, including but not limited to cathode family selection, doping strategy, phase stability, calcination conditions, and electrochemical performance. This implies that cathode design can be better understood as a sequential decision-making problem rather than solely as a static prediction task.

RL provides a natural framework for such sequential optimisation. Contrary to the most supervised machine-learning models used in cathode informatics which are static predictors, RL determines how a candidate should be modified after an unsuccessful result. Cathode development is a path-dependent strategy in which a decision affects the appropriate synthesis conditions, structural evolution, and subsequent experimental choices. Moreover, some consequences of a design action, such as phase instability or voltage decay, may only become apparent after several later evaluation steps. These characteristics motivate a sequential decision-making framework. Therefore, RL involves an agent that interacts with an environment by taking actions, observing outcomes, and updating its policy to maximise cumulative reward. In the context of cathode material design, the state may represent the current material composition, crystal structure, synthesis condition, morphology, interface descriptor, or accumulated experimental history. The action may involve changing an element fraction, substituting a dopant, selecting a precursor, changing calcination temperature, or selecting the next experiment. The reward can be defined using cathode relevant objectives such as high voltage, high reversible capacity, fast ion diffusion, low cost, reduced critical-metal content, improved safety, and improved synthesis ability. In this way, RL can enhance the techno-economic viability of cathode development by reducing reliance on costly and time-consuming trial-and-error experimentation. By learning from previous computational and experimental outcomes, RL can guide the selection of the next most informative design action, avoid repeatedly exploring unpromising candidates and synthesis conditions with favourable performance-to-cost trade-offs. Through multi-objective reward functions, RL can simultaneously consider electrochemical performance, material cost, critical-metal dependence, energy consumption, supply-chain risks, and sustainability, thereby directing resources toward cathode candidates with greater potential for practical and economical deployment.

Recent RL-based inverse design studies in inorganic materials demonstrate the methodological relevance of this approach. Karpovich et al. in [8] proposed deep RL strategies for inverse inorganic oxide materials design by using specified property and synthesis objectives to guide the search for promising compounds. Although such studies are not always cathode specific, they are highly relevant to cathode discovery because many important cathode families are inorganic oxides, phosphates, fluorophosphates, or compositionally complex solid-state materials. These studies show how material design can be reformulated around states, actions, rewards, and constraints rather than only descriptor property prediction. Similarly, autonomous synthesis platforms and active learning based materials workflows have demonstrated how computation, historical data, ML, robotics, and experimental feedback can be integrated to accelerate inorganic powder synthesis [9]. These closed-loop concepts are directly relevant to future cathode discovery, where computational predictions must ultimately be connected to experimentally synthesis validated materials. Therefore, the role of RL in cathode material design should not be viewed merely as another predictive algorithm. Its value lies in its ability to guide sequential choices under multiple objectives and constraints. A well-formulated RL algorithm can connect cathode chemistry, physics-based descriptors, surrogate property models, synthesis constraints, and experimental feedback. This makes RL particularly suitable for design problems where the best material cannot be identified from a single prediction, but emerges from a sequence of chemically valid and experimentally meaningful decisions.

This review focuses on recently published articles with a particular focus on the scientific contributions made in RL, data-driven cathode discovery, and closed-loop materials optimisation relevant to cathode material design. The publication of this review is timely and necessary because rapid advances over the past decade in reinforcement learning, data-driven discovery, and closed-loop materials optimisation have not yet been significantly integrated into a cathode-focused sequential decision-making framework. The necessity of an RL perspective arises from the path-dependent character of cathode discovery. A predictive model may estimate the voltage, stability, or capacity of a candidate, but it does not ordinarily determine whether the next step should be to change the transition-metal ratio, introduce a dopant, modify the calcination conditions, alter the structural prototype, or perform a higher-fidelity experiment. The usefulness of each action depends on the current candidate, the preceding design history, the available evaluation budget, and the outcomes of earlier calculations or experiments. Cathode discovery is therefore not only a mapping from material descriptors to properties; it is a sequence of conditional decisions under competing objectives and physical constraints. Addressing how cathode design can be reformulated as a sequential problem, the review maps composition, interface engineering, and experimental validation onto the RL concepts of state, action, reward, policy, environment, and feedback. It further explains how RL can support physics-informed modelling, data-driven screening, and generative design, while critically identifying barriers involving data availability, reward formulation, physical constraints, and experimental validation. This framework provides the scientific community with a timely roadmap for developing reliable and experimentally actionable RL-guided cathode-discovery strategies.

## 2. Cathode Material Design

Cathode material design is challenging because a change that improves one aspect of performance can easily weaken another. A material with high capacity, for example, may still be unsuitable if it suffers from voltage decay, unstable surfaces, oxygen loss, poor rate capability, safety concerns, or a synthesis route that is difficult to reproduce. Cathode optimisation therefore cannot be reduced to the search for the highest theoretical capacity. It requires a closer understanding of how the material chemistry, crystal structure, processing route, and surface condition shape the behaviour of the full cell [2,3,10].

### 2.1. Major Cathode Materials and Their Design Challenges

Lithium-ion cathodes comprise several chemically distinct families. Among them, layered oxides such as lithium cobalt oxide, denoted as LCO, LiCoO_2_; lithium nickel manganese cobalt oxide, denoted as NMC, LiNi_x_Mn_y_Co_z_O_2_; and lithium nickel cobalt aluminium oxide, denoted as NCA, LiNi_x_Co_y_Al_z_O_2_, where x+y+z=1, are considered the most prominent because of their high energy density and operating voltage. Layered lithium transition-metal oxides contain alternating lithium and transition-metal layers that enable reversible lithium extraction and insertion. For instance, LCO provides high volumetric energy density but is constrained by cobalt cost, thermal stability, and structural degradation at high states of charge. In case of NMC and NCA materials, cobalt is partly replaced with nickel, manganese, or aluminium. Increasing the nickel fraction generally increases practical capacity, but it also promotes lithium–nickel antisite disorder, oxygen release, and electrolyte reactivity [3,11,12].

Spinel and olivine cathodes are found to be more structurally robust alternatives. Spinel cathodes provide three-dimensional lithium-ion transport pathways. Spinel materials such as Lithium nickel manganese oxide, denoted as LNMO, LiNi_0.5_Mn_1.5_O_4_ and Lithium manganese oxide, denoted as LMO, LiMn_2_O_4_ offers comparatively low cost and are prominent materials for fast Li ion transport. Moreover, LNMO operates at a higher potential and is attractive for high-power applications, but it requires stable electrolytes and careful control of transition-metal ordering and surface chemistry. However, their long term operation is still affected by Mn dissolution and high voltage surface reactions [2]. Olivine cathodes such as Lithium iron phosphate, denoted as LFP, LiFePO_4_ and Lithium manganese iron phosphate, denoted as LMFP, LiMn*x*Fe_1−*x*_PO_4_ are equally attractive because of their strong structural and thermal stability. Their major drawback is their modest electronic conductivity, which results from the strong polyanion framework. Therefore, carbon coating and conductive-network design are essential components of material optimisation [10,13].

Sodium-ion cathodes, on the other hand, are promising alternatives because sodium is more abundant and less expensive than lithium. Sodium-ion cathodes, including layered sodium transition metal oxides (Na_*x*_TMO_2_), particularly P2- and O3-type structures; Prussian blue and Prussian blue analogues, and sodium polyanionic and NASICON-type cathodes, each offer distinct practical advantages. Their behaviour reflects the Na content, transition-metal chemistry, and synthesis history [14,15,16,17]. The performance of Na_*x*_TMO_2_ depends on sodium content, transition-metal composition, cation ordering, vacancy concentration, and phase evolution during sodium extraction. Notably, P2 structures provide favourable sodium transport but may undergo high-voltage phase transitions, whereas O3 structures frequently offer higher initial sodium content but can experience complex gliding and multiphase transformations. Likewise, Prussian blue analogues contain open framework structures that support rapid sodium transport and can be synthesised using relatively accessible routes. Sodium polyanionic and NASICON-type cathodes generally provide strong thermal and structural stability, with voltage influenced by the inductive effect of the polyanion group. Their limitations may include modest electronic conductivity, lower volumetric energy density, and the need for conductive coatings or particle-size optimisation.

**Figure 1:**
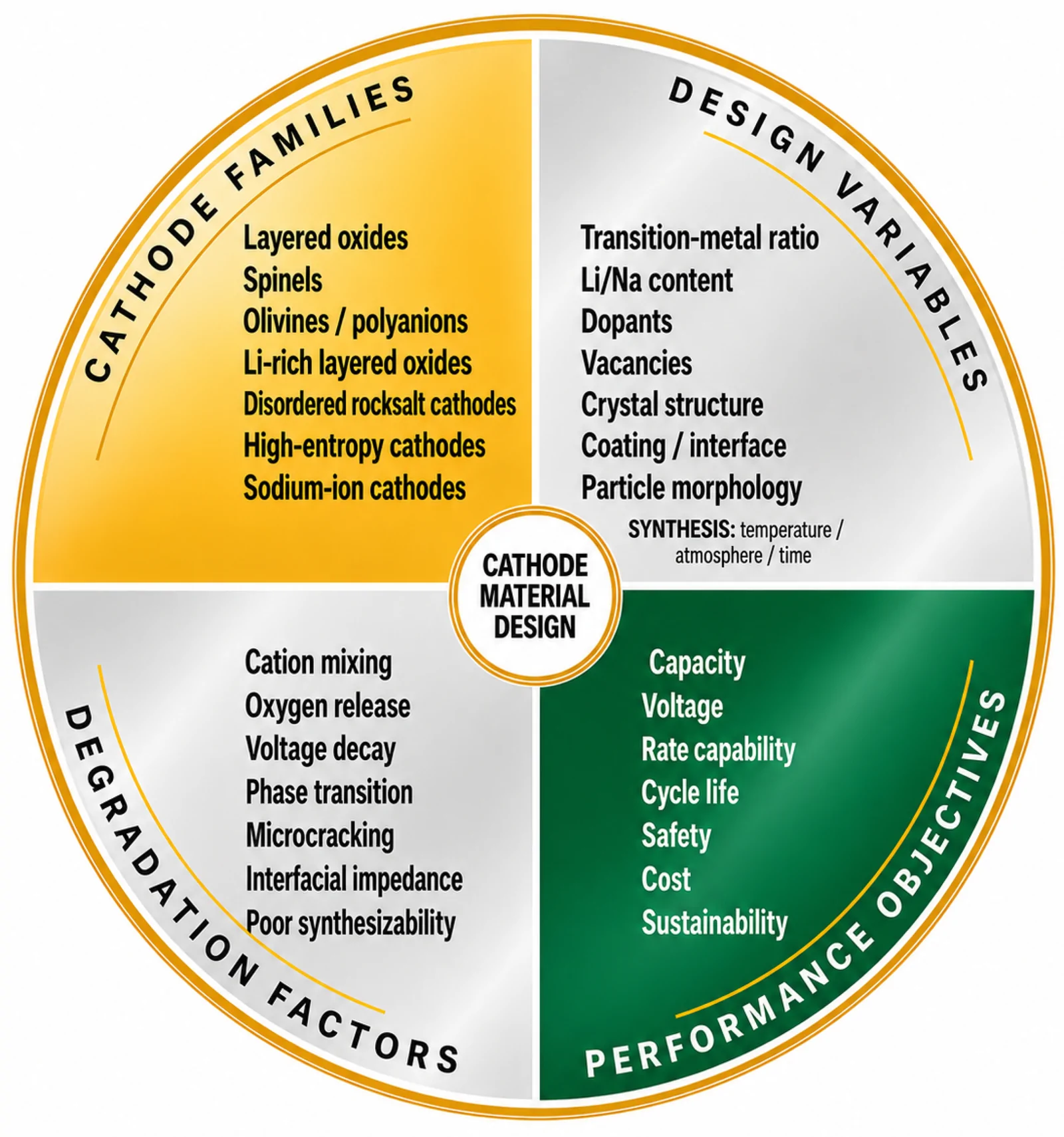
Cathode material design space: families, variables, objectives, and constraints.

Therefore, cathode discovery is not merely a matter of selecting an established chemistry. Rather, it is a design problem in which composition, synthesis, and degradation mechanisms must be considered together. Figure 1 summarises the cathode material design space by connecting the major material families with their key design variables, objectives, and constraints. Table 1 provides the key design variables for different cathode families and their role in the sequential optimisation problems.

### 2.2. Cathode Design Objectives and Performance Constraints

The performance of a cathode cannot be determined on the basis of capacity alone. For instance, a material may exhibit a high theoretical capacity; however, under practical operating conditions, it may suffer from voltage fade, significant oxygen loss, or a synthesis route that is not reproducible at scale. Consequently, cathode optimisation should consider energy density alongside cycling stability, transport behaviour, and long-term material sustainability [13,22].

Balancing these objectives is essential across nearly all cathode families. In Ni-rich layered oxides, increasing the Ni content can enhance capacity while reducing reliance on Co [12,23]. Li-rich layered oxides, on the other hand, pose a different challenge. Oxygen redox provides additional capacity; however, its incomplete reversibility leads to voltage hysteresis and long-term voltage decay [18,24]. In sodium layered oxides, the major challenge is to maintain reversible Na extraction and insertion while minimising phase transitions and volume changes [14,15,16].

Therefore, cathode design is better formulated as a multi-objective optimisation problem rather than as a single-objective maximisation problem:

(1) $$J_{\mathrm{cat}}=f\left(C_{\mathrm{rev}},V_{\mathrm{avg}},E_{\mathrm{stability}},D_{\mathrm{ion}},R_{\mathrm{int}},\Delta V,\Delta V_{\mathrm{cell}},C_{\mathrm{cost}},S_{\mathrm{safety}},S_{\mathrm{sustainability}}\right).$$

Here, $C_{\mathrm{rev}}$ and $V_{\mathrm{avg}}$ describe capacity and average voltage. The terms $E_{\mathrm{stability}}$, $D_{\mathrm{ion}}$, and $R_{\mathrm{int}}$ represent stability, ion transport, and interfacial behaviour, while ΔV captures voltage hysteresis or voltage decay. The remaining terms account for cell-level instability, cost, safety, and sustainability. This expression is not proposed as a universal equation for all cathodes. It is used here to show that the design objective depends on the chemistry, intended application, and relative weight assigned to each constraint.

The consequence is that a useful design action cannot be assessed in isolation. For instance, coating may improve interfacial protection while changing ion transport. Similarly, a dopant can stabilise the structure, although it may also reduce the amount of redox-active material. Also, vacancy engineering in sodium layered oxides can improve phase stability, however, the practical redox capacity must still be preserved. Interestingly, these material-action relationships are important for the RL because they indicate how reward terms reflect both performance improvement and the penalties introduced by a design choice. Table 2 summarises the main cathode design objectives together with the design levers used to achieve them. It highlights that each performance improvement is accompanied by constraints involving stability, transport, synthesis, scalability, cost, or energy density.

### 2.3. Conventional, Computational, and Data Driven Strategies

Cathode optimisation has traditionally relied on a cycle of chemical reasoning, synthesis, characterisation, and electrochemical testing. The emphasis changes with the cathode family. For layered oxides, the main concern is to retain capacity while slowing the structural and interfacial degradation that appears at high states of charge. This is why composition control, particle design, and surface protection are often treated together rather than as separate optimisation steps [3,11]. In olivine and polyanionic cathodes, the crystal framework is generally more stable, but transport limitations make processing and conductive network design central to practical performance [2,12]. Sodium layered oxides introduce a different problem, where small changes in transition metal chemistry, vacancy concentration, or synthesis route can strongly affect phase stability, air sensitivity, and electrolyte compatibility [14,15,16].

Computational screening has significantly reduced the extent of trial and error involved in cathode optimisation. DFT and high-throughput calculations are widely employed to predict properties such as voltage, ion-migration barriers, and oxygen-redox behaviour before experimental synthesis. These tools are valuable for narrowing the candidate space, although the cost becomes significant when disorder, defects, or several transition metals must be considered at the same time. Machine learning has therefore become useful as a surrogate layer, where trained models approximate expensive calculations or experiments and allow faster screening of battery electrode materials [7,27,28,29].

The quality of this approach depends on the data available for cathode materials. Huang et al. showed that battery material information can be extracted from the literature and organised into an auto generated database for materials discovery [30]. Gou et al. developed a similar data extraction route for sodium layered cathodes, linking composition and synthesis records with electrochemical performance [17]. This kind of structured information is important for future RL work because a sequential design model needs to know not only how a material performed, but also what was made, how it was processed, and what outcome followed. Liow et al. demonstrated this connection more directly by using ML assisted synthesis to relate processing conditions to the electrochemical behaviour of LIB cathodes [6]. These studies indicate that cathode research is gradually shifting from isolated property prediction toward more organised design workflows.

The remaining limitation is that most ML based cathode studies still stop at prediction. A model may estimate voltage or rank a candidate, but it does not usually decide the next experimental or computational step. For example, after an unsatisfactory result, a predictive model alone does not determine whether the next action should be to change the synthesis temperature, adjust the dopant level, or modify the surface treatment. This distinction between prediction and action is the point at which RL becomes relevant. In an RL based workflow, ML or DFT models can still provide part of the environment, but the main task is to choose useful design actions over a sequence of decisions. Table 3 summarises representative computational and data-driven methods used in cathode design. It also identifies the main gap between prediction-based models and sequential optimisation, namely the limited ability of current methods to select the next design action.

### 2.4. Cathode Design as a Sequential Decision-Making Problem

Cathode design may begin with a broad range of candidate chemistries. However, the search rapidly becomes conditional and constrained. Each proposed composition must be chemically plausible, synthesizable, and capable of meeting the required voltage and cycling-performance targets. When an outcome is unsatisfactory, the next decision should account for the conditions and results of the preceding trials, including failed experiments or calculations. The researcher or autonomous agent may then adjust the composition, modify the synthesis route, change the processing conditions, or examine a different structural variant. Cathode design is therefore more than a screening problem; it is a sequence of decisions in which each result changes the direction of the next experiment or calculation.

This design history can be written conceptually as

(2) $$H_{t}=\left\{x_{0},a_{0},y_{0},x_{1},a_{1},y_{1},\ldots ,x_{t}\right\},$$

where $x_{t}$ represents state of cathode design at step *t*, $a_{t}$ is the design action taken, and $y_{t}$ is the predicted outcome. In a conventional workflow, the next action is usually chosen through expert judgement, trial-and-error synthesis, or separate computational screening. A RL workflow keeps the same practical sequence, but formalises the choice of the next action through a policy that uses the previous design history and a reward function linked to cathode performance.

**Figure 2:**
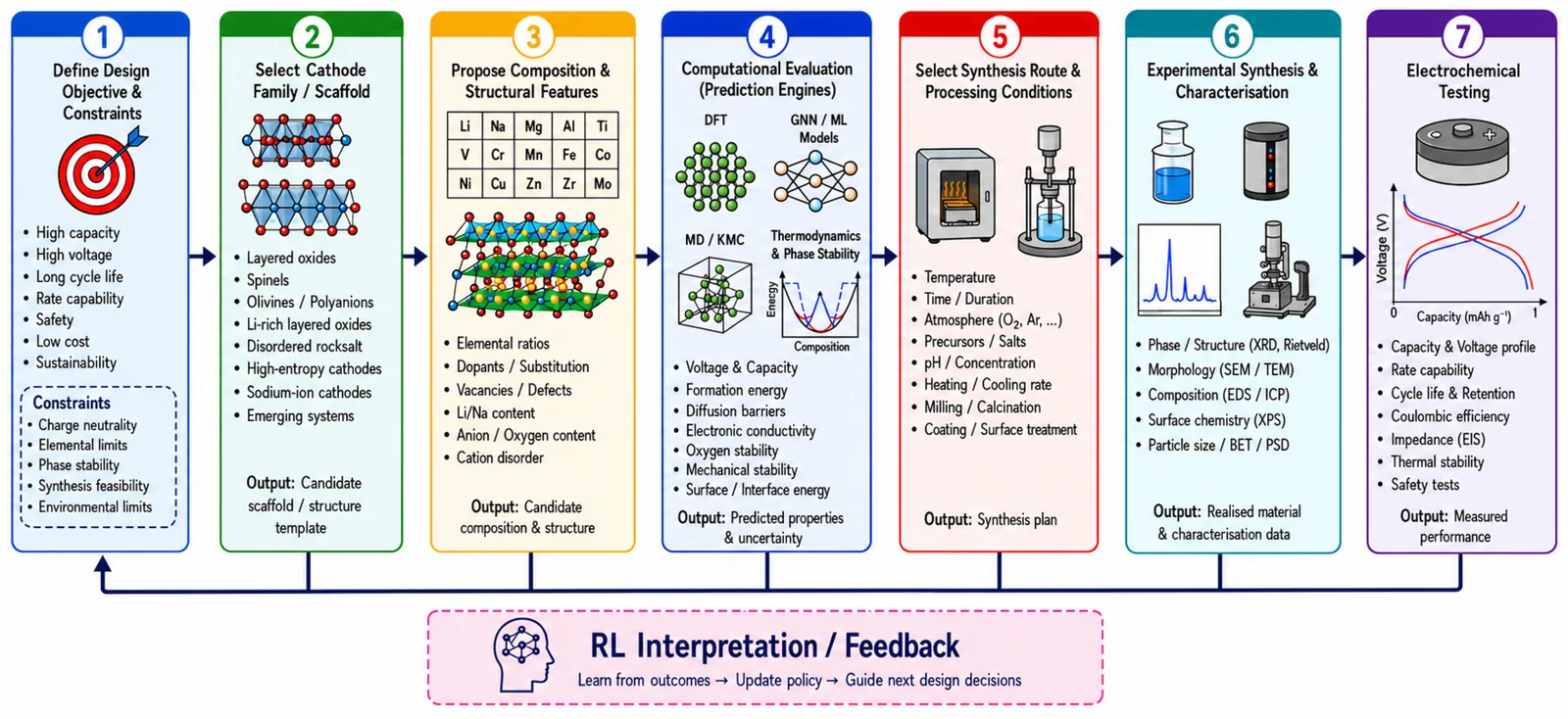
Sequential cathode material design as a RL problem.

In cathode discovery, the design state may contain information about the current chemistry, structure, processing route, surface condition, and available test results. The next action could be a change in composition, synthesis, defect design, coating, or experimental selection. The feedback may come from calculation or experiment, depending on whether the workflow is computational, laboratory based, or closed loop. This framing is important for Section 3, where RL is treated not as a general artificial intelligence label, but as a way to decide what cathode design step should be taken next. Figure 2 illustrates cathode material design as a sequential reinforcement-learning problem in which the current state determines the next design action and resulting feedback. The updated state and accumulated design history then guide subsequent decisions towards the required cathode performance objectives.

## 3. RL for Cathode Material Design

As cathode candidate is shaped by linked choices in chemistry, structure, and performance evaluation, and each choice affects each other. RL is useful in this context because it treats design as a sequence of decisions rather than as a single prediction problem. A static machine learning model can estimate a property from descriptors, but an RL model is concerned with what action should be taken next after a result is observed.

Most conventional supervised ML methods used in cathode design learn a static relationship between material descriptors and target properties, enabling candidate prediction, screening, or ranking. In contrast, RL learns a policy for selecting the next design action based on the current state and the outcomes of previous actions. This enables RL to account for path-dependent decisions, delayed consequences, cumulative multi-objective rewards, and adaptive exploration of the design space. Conventional ML models may still be used as surrogate predictors within the RL environment, but they do not independently determine the sequence of composition, structure, synthesis, or experimental modifications required to reach a design objective.

### 3.1. RL Concepts for Cathode Discovery

In RL, an agent improves its decisions by interacting with an environment. At step *t*, the agent observes a state $s_{t}$, chooses an action $a_{t}$ according to a policy $\pi (a_{t}|s_{t})$, receives a reward $r_{t}$, and moves to a new state $s_{t+1}$. The objective is to learn a policy that maximises the expected cumulative reward, which can be presented as

(3) $$J\left(\pi \right)=E_{\pi }\left[\sum_{t=0}^{T}\gamma^{t}r_{t}\right]$$

where $E_{\pi }$ denotes the expected value of the cumulative discounted reward over all possible trajectories generated by the policy π, accounting for uncertainty in the agent’s actions and environment transitions. Here, γ denotes the discount factor and *T* represents the design horizon [33]. In cathode design, this horizon may be interpreted as the number of experimental or computational decisions made before a candidate is accepted, rejected, or redesigned.

The key conceptual shift is that a cathode candidate is no longer regarded as a fixed entity in a database; rather, it is treated as an evolving design state. An action may modify the candidate’s chemistry, crystal structure, or synthesis conditions. The resulting outcome can be obtained from computation, experiment, or a surrogate model [34]. Importantly, this outcome is not treated merely as an additional data point; instead, it is used to guide the subsequent design decision [35].

This differentiates RL from ordinary inverse screening. A supervised model can predict voltage, stability, migration behaviour, or capacity, but it does not decide how a candidate should be changed after an unsatisfactory result [36,37]. This decision layer is added by the RL. Early RL work in molecular design is useful for this review because it showed how candidate structures can be generated, modified, scored, and improved through repeated policy updates [38]. For cathode discovery, the same idea has to be adapted to solid-state compositions, periodic crystal structures, synthesis conditions, and electrochemical feedback [34,39].

A cathode-oriented reward can be written in a general form as

(4) $$\begin{array}{ccc} R_{\mathrm{cat}} & = & w_{1}C_{\mathrm{rev}}+w_{2}V_{\mathrm{avg}}-w_{3}E_{\mathrm{hull}}\\ & & -w_{4}E_{\mathrm{mig}}-w_{5}\Delta V_{\mathrm{decay}}-w_{6}P_{O}\\ & & -w_{7}R_{\mathrm{int}}-w_{8}C_{\mathrm{critical}}-w_{9}P_{\mathrm{synth}}. \end{array}$$

Here, $C_{\mathrm{rev}}$ and $V_{\mathrm{avg}}$ reward reversible capacity and average voltage. The terms $E_{\mathrm{hull}}$, $E_{\mathrm{mig}}$, $\Delta V_{\mathrm{decay}}$, $P_{O}$, and $R_{\mathrm{int}}$ penalise instability, difficult ion migration, voltage decay, oxygen-related degradation, and interfacial resistance. The final terms $C_{\mathrm{critical}}$ and $P_{\mathrm{synth}}$ account for critical element dependence and poor synthesisability. In practice, these quantities cannot be added in their raw physical units. Each term would first need to be normalised or converted into a dimensionless score before the weights are applied. The expression is therefore not a universal reward function. It is a template showing how cathode design objectives can be translated into RL terms, with the weights adjusted according to the chemistry, application, and constraints of the target cathode system. Table 4 organises representative RL studies into foundations, composition and structure applications, and synthesis and interface applications relevant to cathode discovery. It compares the contribution and cathode relevance of each method while identifying the boundaries and limitations of the available evidence.

**Figure 3:**
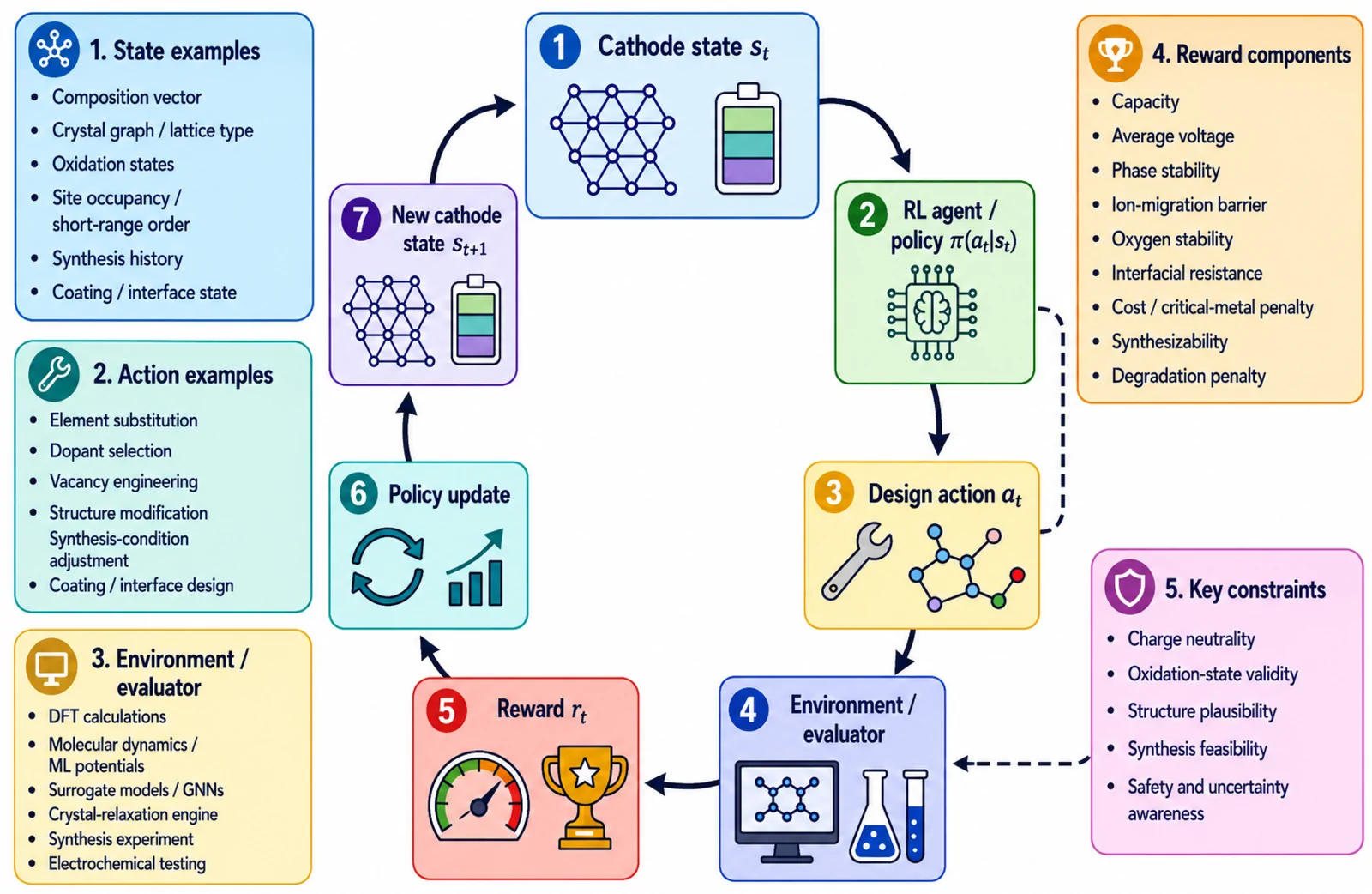
RL formulation of cathode material discovery.

Figure 3 presents the RL formulation of cathode material discovery by connecting the material state, available design actions, environment response, reward, and policy. It illustrates how repeated feedback can guide cathode discovery as a sequential optimisation process rather than as a single property prediction task.

### 3.2. Mapping of RL Objects to Cathode Discovery

The primary objective when applying RL to cathode discovery is to define the RL components from a materials science perspective. It is worth noting that a weak formulation might represent the state solely as a chemical formula and define the reward only in terms of capacity. Such a formulation would be too narrow for cathode design, particularly because performance depends on composition, synthesis conditions, interfacial behaviour, and degradation mechanisms [33,34]. Consequently, a reliable RL formulation must describe each candidate in sufficient detail to enable the agent to make chemically meaningful decisions.

The state should therefore represent the current cathode candidate along with all information about it. For a composition centred workflow, this can include elemental fractions, redox-active species, oxidation-state balance, and critical-element content. For a structure centred workflow, the state must carry structural information such as site occupancy, local coordination, diffusion pathways, and disorder. When synthesis or interface design is part of the problem, the state should also record processing history, phase purity information, surface condition, and electrolyte compatibility. In this way, the cathode is converted from a static material label into a decision state that can evolve as new calculations or experiments are performed, consistent with emerging RL formulations for materials and crystal design [34,44,47].

The action space defines the changes that the agent is allowed to make. In some studies, this may mean changing the transition-metal ratio, replacing a critical element, adding excess Li or Na, or introducing a dopant. In others, the action may involve changing a structural prototype, creating a vacancy, modifying site occupancy, or relaxing a generated structure. For workflows closer to experiment, the action can also refer to synthesis temperature, atmosphere, holding time, precursor choice, or surface treatment. The exact action set should not be chosen only for algorithmic convenience. It has to reflect what is chemically feasible and experimentally meaningful for the cathode family being studied.

Crystallographic constraints should be incorporated through both the action space and the reward function. It ensures that generated cathode candidates remain physically plausible. The action space can be restricted to modifications that preserve charge neutrality, valid elemental oxidation states, allowable site occupancies, appropriate coordination environments, and crystallographic symmetry. Periodic boundary conditions should be enforced through periodic crystal representations and structure relaxation procedures, while actions that create atomic overlaps, unrealistic bond lengths, invalid stoichiometries, or over occupied lattice sites should be rejected. Remaining deviations from preferred coordination, structural stability, or site occupancy can be included as penalty terms in the reward function. Thus, hard constraints prevent invalid candidates from being generated, whereas soft reward penalties guide the agent toward chemically and crystallographically realistic structures.

The reward defines what the agent is encouraged to improve. For cathode materials, a reward based only on capacity would be scientifically weak, because high capacity can be accompanied by voltage decay, oxygen loss, poor transport, high cost, or poor synthesis feasibility. Reward design is therefore one of the main scientific decisions in cathode RL. Multi-objective studies show why viability, diversity, synthesisability, and property trade-offs must be represented rather than collapsed into an incomplete target [37,48,49]. The reward determines whether the agent searches for realistic cathode candidates or simply exploits an incomplete numerical target.

The remaining RL objects follow from these choices. The policy describes how the agent selects the next design move from the current state. The environment provides the response to that move, either through calculation, surrogate prediction, experimental testing, or a closed-loop combination of these. Feedback updates the design history, while constraints prevent chemically invalid, unsafe, or impractical candidates from being rewarded [33,38,41]. A cathode-specific RL framework is therefore not defined only by the algorithm used. It is defined by how carefully the material state, allowed actions, reward terms, environment response, and constraints are matched to the cathode problem.

**Figure 4:**
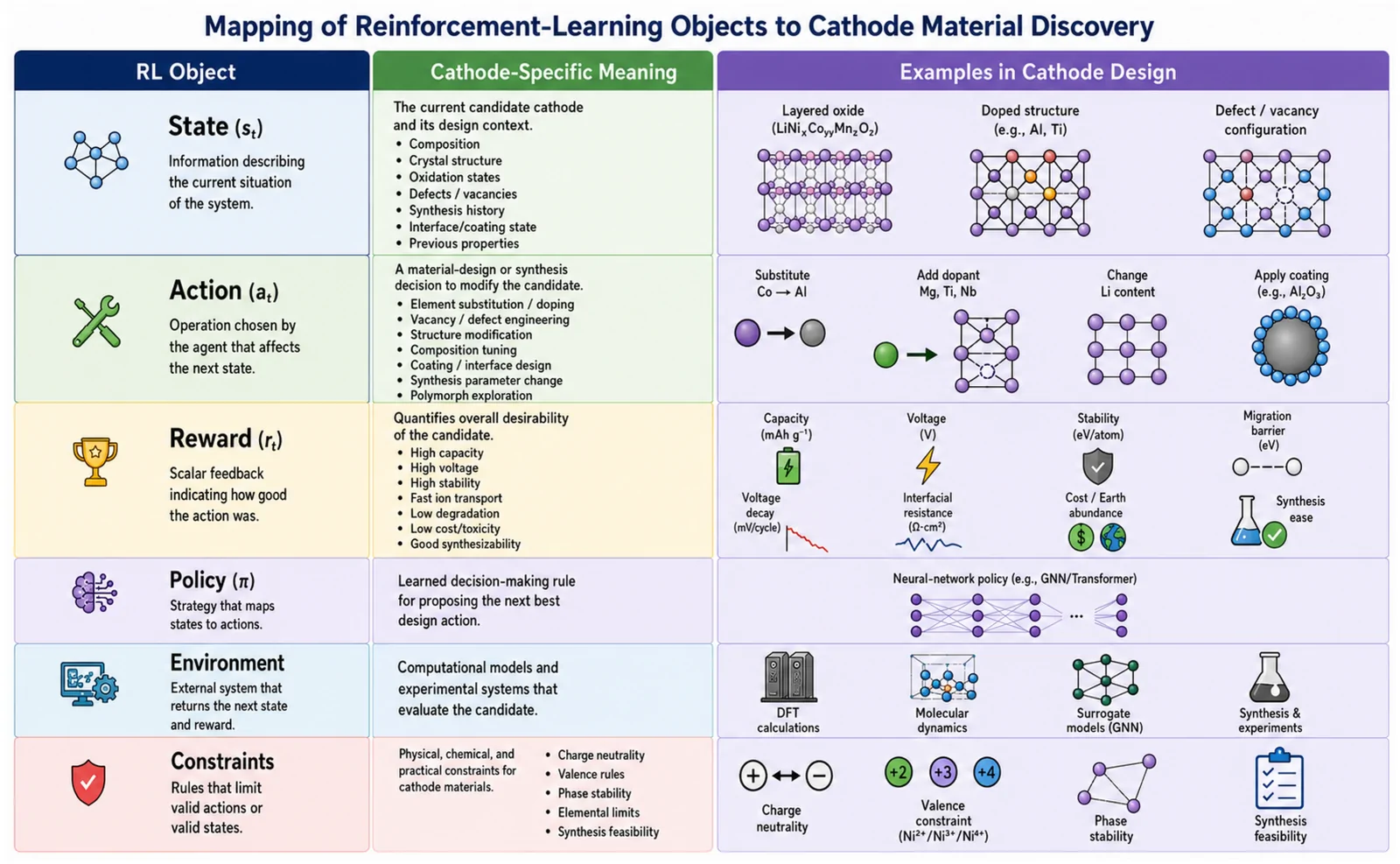
Mapping of RL objects to cathode material discovery.

Figure 4 maps the principal RL objects, including the state, action, reward, policy, environment, feedback, and constraints, onto cathode material discovery. It shows that a chemically meaningful RL framework depends on representing material information, feasible design actions, and realistic evaluation criteria rather than only selecting an RL algorithm.

### 3.3. RL Guided Composition and Structure Generation

A natural entry point for applying RL to cathode design is composition-space exploration. However, this should not be treated as unconstrained editing of chemical formulas. Instead, an agent may begin with a partially specified composition or a parent cathode family and propose chemically valid modifications. The reward can then reflect the expected electrochemical value while penalising reliance on critical elements, thermodynamic instability, poor synthesisability, and other practical limitations. This approach is analogous to molecular RL, in which candidates are improved through a set of permitted operations and evaluated using property-based feedback [35,36,38,39].

Structure generation is a more stringent problem because materials with the same composition may behave differently depending on atomic ordering, diffusion topology, and local coordination. These structural features influence voltage, phase evolution, and degradation. A structure-level RL agent must therefore operate on lattice sites, crystal graphs, or descriptors derived from relaxed structures. Recent studies on RL related to crystal structure prediction, and inorganic structure generation interpret how structural states, site based actions, and relaxation feedback can be incorporated into a sequential search workflow, even though these studies are not cathode specific [44,45,46,47,48,49].

### 3.4. RL Guided Synthesis and Closed Loop Optimisation

A cathode design is meaningful only when it can be synthesised reproducibly. Therefore, synthesis should not be treated merely as a final validation step following composition or structure screening. Rather, it should be regarded as an integral part of the design problem itself. A composition that appears promising on the basis of calculated voltage or stability may still fail experimentally if it produces impurity phases or particles with poor electrochemical behaviour. RL is particularly useful in this context because it can learn from the outcome of each synthesis attempt and use that information to guide the selection of subsequent synthesis routes, as demonstrated more broadly in synthesis-optimisation studies [52,53,54].

A synthesis aware RL framework not only judges whether the required composition is achieved, but also by whether the obtained material has acceptable morphology, phase purity, and electrochemical activity. Offline RL, and reaction-condition optimisation can provide methodological precedents for this cathode-specific extension, although they are not directly focused on battery cathodes [53,55,56,57].

A closed-loop cathode-discovery strategy can be represented as

(5) $$s_{t}\to a_{t}\to y_{t}\to r_{t}\to \pi_{t+1}.$$

Here, $s_{t}$, $a_{t}$, and $y_{t}$ denote the current design state, the selected design action, and the outcome returned by a calculation or experiment, respectively. Meanwhile, $r_{t}$ represents the reward assigned to that outcome, and $\pi_{t+1}$ denotes the updated policy used to guide the next decision. It is worth noting that unsuccessful trials are not wasted. An unsuccessful synthesis route or an unstable structure still provides information that can influence the subsequent decision. Consequently, RL transforms cathode optimisation into an adaptive learning process rather than a sequence of disconnected predictions and experiments [53,54].

### 3.5. RL Algorithm for Cathode Design

There is no single RL algorithm that is suitable for all cathode design problems. The choice depends on how the material problem is represented and how much freedom the agent has been given. If the agent works within a small set of chemically valid moves, such as changing an element, choosing a dopant, or introducing a defect, value based methods such as Q learning and deep Q networks can be appropriate, as illustrated by discrete molecular-edit optimisation with deep Q-learning [39]. Their advantage is that the action space is easier to control. Their limitation is that the environment must already know enough chemistry to reject unrealistic moves. Otherwise, the agent may learn numerical shortcuts instead of useful cathode design rules.

More flexible algorithms are needed when the design cannot be reduced to a small menu of actions. Policy gradient and actor critic methods are better suited to problems where the agent must generate candidates, explore uncertain choices, or handle partly continuous variables [35,38,61]. This is relevant for organic cathodes, coating molecules, crystal graph construction, and target conditioned structure generation. These methods give the agent greater freedom, but they also make reward design more delicate. If the reward is poorly scaled or the surrogate model is weak, the agent can optimise the score without producing a material that is chemically realistic or experimentally useful. Table 5 compares RL algorithm families according to their suitable cathode-design tasks, typical action spaces, quantitative selection criteria, and primary limitations. It provides evidence-based guidance for selecting an algorithm according to the action-space structure, data availability, evaluation cost, and optimisation objectives.

Cathode discovery also involves changing targets. A high voltage layered oxide, a low cost sodium cathode, a stable organic electrode, and a fast ion transport coating are not judged by the same priorities. Goal conditioned RL is useful in this setting because the desired property profile can be supplied to the agent as part of the input, an approach demonstrated in goal-conditioned inverse design [62]. The policy can then adapt to different design aims rather than being trained from the beginning for every new cathode objective. This is particularly relevant when the balance between voltage, cost, stability, transport, and sustainability changes with the intended application.

**Figure 5:**
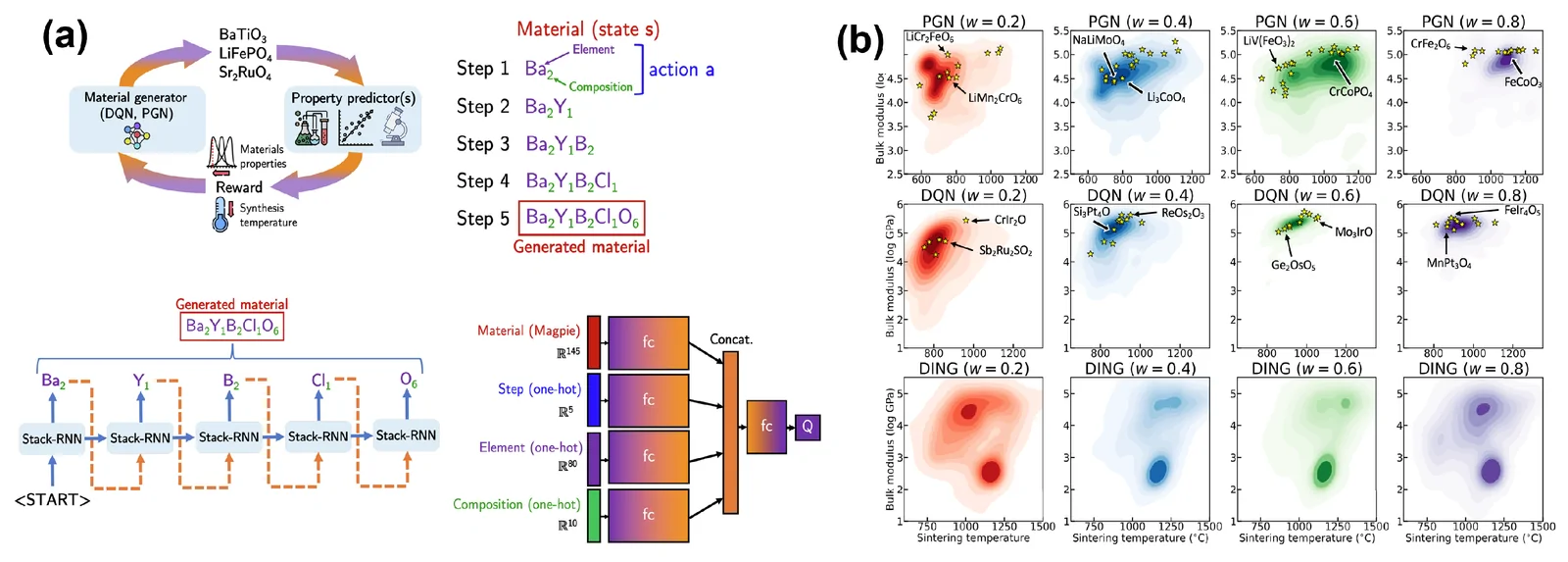
(**a**) RL-based inorganic-material generation workflow and the policy gradient network (PGN) and deep Q-network (DQN) architectures; (**b**) Multi-objective property distributions generated by PGN, DQN, and DING under different synthesis–property reward weights; stars indicate near-Pareto-optimal compositions. Reproduced with permission [8].

The available evidence usually comes from published compositions, synthesis routes, and electrochemical results, rather than from continuous interaction between an agent and a real experimental platform. Historical data can therefore be used to initialise a policy before new experiments are attempted. For instance, LFP may be used as a representative cathode system to illustrate the proposed RL framework as presented in the Figure 5. In the workflow reported by Karpovich et al., LFP is shown as an example composition within a feedback loop in which an RL-based material generator proposes inorganic compounds and receives rewards from predicted material and synthesis properties. The state is represented by the evolving material composition, while actions correspond to sequentially adding elements and their stoichiometric coefficients. The same study further demonstrates multi-objective RL using weighted synthesis and property rewards to identify near-Pareto-optimal compositions. Although LFP itself was not optimised as a dedicated case study, this formulation provides a transferable basis for focused LFP optimisation involving composition, dopants, synthesis conditions, and competing performance processing objectives.

Section 3 treats RL as a decision-making layer for cathode material discovery, not as a replacement for cathode chemistry, DFT, synthesis, or electrochemical testing. Its value is in linking these tools into a workflow where each calculation or experiment informs the next design step. In this design, the cathode candidate possesses its design history. Each modification is assessed against synthesisability, electrochemical performance, and stability. Subsequently, the outcome is then used to refine the next decision. In this way, this reinforces the main argument of this review: cathode material design is not only a prediction or screening task, but a sequential decision-making problem.

## 4. Challenges and Possible Solutions

RL can support cathode discovery only when its decisions are based on the comparable data, chemical validity and experimental feedback. It should select the next useful action rather than replacing physical modelling. With respect to RL implementation, the core challenges are based on the biased reward functions and weak model evaluation. The unreliable evaluation can push unsuitable candidates towards design leading to less efficient and effective material. In this section, core challenges associated with RL implementation in the cathode discovery have been discussed along with their possible solutions.

### 4.1. Data Bias

The Cathode datasets rarely link a parent candidate, design action, and outcome. Data-scarce LIB research therefore requires targeted mitigation of these design variables [64]. Failed syntheses and negative electrochemical results should be retained to reduce data bias [65]. Since protocol inconsistency can distort the apparent reward functions so voltage window, electrolyte, and cell format must be considered in RL formulation [66]. Structured partial records can still guide inorganic design when combined with physical filters [67]. Foundation models may provide transferable representations, but domain shifts across cathode chemistry can make out-of-order-distortion predictions unreliable [68]. Consistent data splits, reward definitions, and evaluation criteria are needed for a comprehensive implementation of RL algorithms [69]. In this regard, temporal splits are preferable because random splits may place closely related compositions in both training and test sets.

### 4.2. Rewards, Constraints, and Delayed Outcomes

Another challenge in implementing RL for cathode discovery is designing an appropriate reward function. Properly conditioned inorganic generation offers one way to manage these competing objectives. Before any of that matters, a feasibility gate has to reject candidates that violate charge neutrality or plausible oxidation states [70]. Energy above the hull should then be combined with metastability and synthesis. Since accessible materials are not always in ground states. Similarly, delayed credit is another challenge. A design action may look beneficial at first and only cause phase change or capacity loss after extended cycling. In this case, intermediate mechanistic signals and final cycling outcomes need to stay distinguishable. Reward priorities also shift as the search moves from discovery to optimisation. Fixed weights do not survive that transition and they let whichever property is easiest to predict quietly take over.

### 4.3. Multi-Fidelity and Evaluations

RL should decide which candidate are to be evaluated with respect to their fidelity rate. The pipeline typically starts with generative crystal models proposing candidates which further becomes the foundation atomic models that can screen before anyone commits to DFT [71]. Active-learning force fields cut down relaxation cost, and general interatomic potentials can be fine tuned for a specific cathode family [72]. Surface and interface accuracy remain uneven, and that unevenness is exactly where the simulation-to-real gap for coatings and degradation show up. GNNs add rapid energy material descriptors, while cathode specific transformers contribute redox or voltage components [73,74]. Moreover, high fidelity failures have to update both the surrogate and the policy. Otherwise, the agent will keep exploiting a proxy it already knows is wrong.

### 4.4. Safe and Reproducible Closed Loops

A candidate should enter the loop with a plausible synthesis route and leave only after structural and electrochemical validation [75]. Getting there requires stopping criteria, versioned evidence, reproducible protocols, and a safe operating envelope at every stage of the loop. In such cases the explainable models cannot be correctly adopted and may require comprehensive design considerations. Explainable models can surface the variables tied to cathode thermal degradation, and mechanistic models can connect predictions back to the underlying electrochemical processes [76,77]. Out of domain or sensitive experiments may still need expert guided review before anyone synthesises them and draw conclusions.

## 5. Conclusions

Cathode material design remains difficult because strong performance in one measure does not guarantee a useful battery material. A cathode with attractive capacity or voltage may still fail because it is hard to synthesise, unstable during cycling, or incompatible with the cell environment. This review has argued that such problems are better handled as linked design decisions rather than isolated prediction tasks. In this context, RL provides a useful way to describe cathode discovery as a process in which each calculation, synthesis attempt, or electrochemical result influences the next design choice.

The practical use of RL for cathode discovery will depend on how well it is grounded in cathode chemistry and experimental evidence. Current limitations include incomplete datasets, weak reporting of failed trials, uncertain reward design, and the risk of generating candidates that look promising only in a model. Future work should therefore focus on constrained and experimentally validated RL workflows, where physical rules, synthesis feasibility, degradation behaviour, and uncertainty are considered before a candidate is treated as successful. Used in this way, RL can support more adaptive cathode discovery without replacing the chemical knowledge, simulations, and experiments needed to make the materials real.

## Figures and Tables

**Table 1:** Representative cathode systems classified according to their crystal-chemical family and dominant design variables or trade-offs relevant to sequential optimisation.

Cathode Family/Design Space	Key Design Variables or Trade-Offs	Role in Sequential Optimisation	Ref.
Layered, spinel, olivine, and polyanion LIB cathodes	Chemistry governs voltage, capacity, stability, and cost.	Select the initial cathode family.	[10]
Layered oxides, spinels, and polyanionic cathodes	Class-specific structural, thermal, and electrochemical limitations.	Set family-specific rewards and constraints.	[2]
Ni-rich layered NMC/NCA cathodes	Ni content, crystallinity, coating, synthesis route, and degradation.	Optimise composition, morphology, interface, and processing.	[3]
Ni-rich NCM cathodes	High capacity versus structural and interfacial stability.	Apply a multi-objective reward that penalises degradation.	[11]
Li-rich layered oxide cathodes	Anionic-redox capacity versus oxygen loss and voltage hysteresis.	Constrain oxygen-redox reversibility and voltage hysteresis.	[18]
Co-free Li-rich layered oxide	Cation ordering and structural design reduce voltage decay.	Optimise structural sequence and cation arrangement.	[19]
Li-excess cation-disordered rocksalt cathodes	Li excess, oxygen redox, and 0-TM percolation.	Tune cation ratio and Li excess subject to percolation constraints.	[20]
High-entropy DRX cathodes	Multi-element composition and entropy effects.	Expand actions to element selection and entropy-guided composition.	[21]
Sodium layered oxide cathodes	P2/O3 phase, Na content, and transition-metal ratio.	Define a Na-ion-specific composition and phase search space.	[14]
Vacancy-engineered P2 sodium layered cathode	Transition-metal vacancies suppress phase transitions and improve stability.	Treat vacancy engineering as a sequential design action.	[15]

DRX = disordered rocksalt; LIB = lithium-ion battery; NCA = lithium nickel cobalt aluminium oxide; NCM/NMC = lithium nickel cobalt manganese oxide; P2/O3 = sodium layered-oxide stacking types; TM = transition metal.

**Table 2:** Representative cathode design objectives, design levers, and associated trade-offs.

Objective	Design Lever or Insight	Constraint or Trade-Off	Study
Long-life Ni-rich NCMA cathode	Al incorporation and quaternary composition improve mechanical and cycling stability.	Compositional complexity, synthesis control, and phase homogeneity.	[25]
Cathode–electrolyte surface stability	Identifies electrolyte-decomposition pathways at Ni-rich cathode surfaces.	High voltage increases interfacial reactivity and safety risk.	[12]
Disorder-driven degradation	Models cation-disorder formation and degradation in layered oxides.	Narrower voltage windows limit degradation but reduce usable energy.	[23]
Voltage-decay mitigation	Co-free Li-rich design maintains high capacity while reducing voltage decay.	Oxygen redox and structural evolution require careful control.	[19]
O2/O3 Li-rich structural design	Compares structural routes for inhibiting voltage decay.	Transition-metal migration must be suppressed without excessive capacity loss.	[24]
Coating transport properties	Machine-learned interatomic potentials assess Li-ion conduction in coatings.	Chemical protection must be balanced against Li-ion transport.	[26]
Cobalt-free battery transition	Maps pathways and constraints for reducing cobalt dependence.	Supply-chain resilience versus performance and manufacturability.	[13]
Long-life sodium layered cathode	Transition-metal vacancies suppress phase transitions and improve thermal and structural stability.	Redox activity and practical energy density must be preserved.	[15]
High-energy O3-type Na cathode	Reconstruction-layer design improves cycling stability and Na-ion distribution.	Added synthesis complexity and scalability constraints.	[16]
Sodium-ion techno-economic roadmap	Links capacity, voltage, electrode thickness, and Ni content to cost and energy density.	Material gains must yield cell-level techno-economic benefits.	[22]

NCMA = lithium nickel cobalt manganese aluminium oxide; O2/O3 = layered-oxide stacking types.

**Table 3:** Representative computational and data-driven resources for cathode design and their gaps for sequential optimisation.

Method and Target	Data or Model Output	Sequential-Design Gap	Study
Literature mining; battery-material database	Capacity, voltage, and energy-density records extracted from the literature.	Data cleaning, provenance, and experimental context are required for decision models.	[30]
NLP extraction; sodium layered cathodes	Composition, synthesis, and performance records mined from the literature.	Missing context and extraction errors may bias downstream optimisation.	[17]
ML voltage prediction; metal-ion electrodes	Surrogate voltage models for high-throughput electrode screening.	Predicts and ranks candidates but does not select synthesis or modification sequences.	[27]
ML screening; metal-ion electrodes	Candidate ranking from computed and materials-database descriptors.	Does not map composition choices to sequential synthesis decisions.	[29]
Attention-based GNN; electrode voltage	Structure-aware model for average-voltage prediction.	Requires stability, cost, transport, and synthesisability constraints.	[28]
Voltage mining and XGBoost; Li-ion cathodes	Chemistry-based voltage surrogate derived from combined calculated datasets.	Voltage-only rewards omit stability, transport, cost, and synthesis.	[7]
ML-assisted synthesis; LIB cathodes	Links synthesis descriptors with electrochemical performance.	Closed-loop action selection and sequential process control remain limited.	[6]
Microscopy and statistical ML; NCM523 degradation	Relates defect evolution to impedance growth and capacity fading.	Supports reward penalties but does not generate a design policy.	[31]
ML interatomic potentials; coating transport	Li-ion conduction estimates for cathode-coating materials.	Coating selection also requires chemical-stability and manufacturability constraints.	[26]
ML transport models; Li-ion migration and percolation	Datasets and surrogates for migration barriers and percolation descriptors.	Transport outputs must be coupled to redox, phase stability, and cycling performance.	[32]

GNN = graph neural network; LIB = lithium-ion battery; ML = machine learning; NCM523 = ${\mathrm{LiNi}}_{0.5}{\mathrm{Co}}_{0.2}{\mathrm{Mn}}_{0.3}O_{2}$; NLP = natural language processing; XGBoost = extreme gradient boosting.

**Table 4:** RL studies based on foundations, composition/structure, and synthesis/interface applications related to cathode discovery.

RL Method or Application	Main Contribution	Relevance to Cathode Discovery	Limitation	Ref.
**FOUNDATIONS**				
RL foundations	Introduces agent–environment interaction, policies, values, returns, and exploration.	Formalises cathode design as a sequential decision-making problem.	General practitioner textbook; not a materials-research study.	[33]
Sequence-based policy-gradient RL	Fine-tunes a SMILES generator using augmented likelihood and property-based scores.	Supports reward-guided generation of organic cathodes, redox-active molecules, and interphase materials.	Molecular representation; does not describe periodic solids.	[35]
Generative–predictive RL	Couples a SMILES generator with a property predictor in the ReLeaSE framework.	Provides a template for coupling cathode candidate generators with surrogate reward models.	Demonstrated on molecular-property and drug-design tasks.	[36]
Graph-convolutional policy network	Constructs molecular graphs using policy gradients, adversarial objectives, and chemical-valence rules.	Illustrates constrained graph-editing actions for candidate generation.	Molecular validity does not enforce crystal symmetry, periodicity, or solid-state charge balance.	[38]
Deep Q-learning	Uses chemically valid molecular edits, double Q-learning, and multi-objective rewards.	Provides an analogue for substitution, doping, and stoichiometry changes.	Molecular edits are simpler than solid-state structural transformations.	[39]
Quantum-guided RL	Builds molecules in Cartesian coordinates using approximate quantum-energy rewards.	Supports physics-informed reward functions instead of relying only on empirical labels.	Treats isolated molecular systems rather than periodic lattices.	[40]
Forward-synthesis RL	Applies valid chemical reactions to commercially available building blocks during candidate generation.	Supports synthesis-aware design of organic cathodes, redox molecules, and coating materials.	Uses organic reaction templates; does not model inorganic phase formation.	[41]
Sample-efficient molecular RL	Uses augmented hill climbing to reduce scoring-function evaluations.	Relevant when candidate evaluation requires expensive simulations or property models.	Demonstrated primarily on molecular-docking and drug-design tasks.	[42]
RL coupled with active learning	Uses surrogate-assisted acquisition to reduce expensive oracle evaluations.	May reduce the number of DFT calculations or experiments required during cathode search.	Demonstrated with molecular oracles and sensitive to surrogate-model error.	[43]
**COMPOSITION/STRUCTURE**				
Crystal-structure prediction	Learns adaptive move-selection policies during crystal-structure search.	Supports prototype search, cation ordering, and site arrangement in cathodes.	Optimises structural energy rather than electrochemical performance.	[44]
Fixed-cell crystal relaxation	Uses deep RL to select atomic-position updates during structure relaxation.	Relevant after doping, vacancy creation, or site-occupancy changes produce distorted structures.	Tested on Al–Fe structures using embedded-atom-method energy surfaces.	[45]
Online crystal-discovery benchmark	Benchmarks RL using DFT-calculated band gap, bulk modulus, and density.	Provides a framework for cathode benchmarks with expensive reward evaluations.	Does not include voltage, capacity, or degradation objectives.	[46]
Autoregressive crystal generation	Fine-tunes CrystalFormer using stability and material-property predictor rewards.	Supports conditional generation toward stability and cathode-property proxies.	Relies on learned reward models rather than direct experimental feedback.	[47]
RL-guided diffusion generation	Fine-tunes diffusion models for goal-directed and multi-objective crystal design.	Supports optimisation of stability, performance, cost, and supply-chain criteria.	No cathode specific demonstration.	[48]
Diversity-guided crystal generation	Balances novelty, structural diversity, and thermodynamic viability.	Supports exploration of under-sampled cathode chemical spaces.	Not demonstrated specifically on cathode materials.	[49]
Multi-agent crystal optimisation	Treats atoms as cooperating agents that collectively optimise periodic structures.	Supports local relaxation of complex cathode supercells.	Addresses geometry optimisation rather than composition or electrochemical performance.	[50]
Multi-objective DQN	Traverses a material-composition graph using sparse adsorption rewards.	Provides an analogue for discrete cathode composition search under sparse feedback.	Catalyst-focused study; does not generate complete cathode structures.	[51]
Materials RL review	Reviews RL methods, applications, and challenges in materials discovery.	Positions cathode RL within the broader emerging materials-design field.	RL implementation is not primary objective.	[34]
**SYNTHESIS/INTERFACE**				
Reaction-condition optimisation	Selects experimental conditions using accumulated reaction outcomes.	Provides an analogue for optimising temperature, time, atmosphere, and composition.	Demonstrated on microdroplet reactions and Ag nanoparticle synthesis, not cathodes.	[52]
Model-based offline RL	Learns time-dependent CVD synthesis schedules from computational simulations.	Provides a strong analogue for adaptive calcination and phase-formation schedules.	Computational MoS_2_ CVD study; not a physical closed-loop cathode experiment.	[53]
Autonomous-experimentation strategy	Compares AI-guided decision strategies in a single-period RL framework.	Supports experiment selection when cathode synthesis objectives are expensive and competing.	Decision strategies were evaluated using simulated experiments.	[54]
Reaction-route optimisation	Uses a value network to select routes from combined organic and biological reaction networks.	Relevant to precursor and organic-cathode synthesis-route planning.	Does not model reaction yield, selectivity, or solid-state processing.	[55]
Generative reaction optimisation	Generates reactants or catalysts and evaluates predicted yield or selectivity.	Relevant to organic cathodes, coating molecules, and molecular precursors.	Predictor-based organic-reaction study without experimental cathode validation.	[56]
Flow-chemistry control	Applies deep RL to self-optimisation of continuous-flow reaction conditions.	Provides transferable control logic for automated cathode-precursor processing.	Demonstrated on a single imine-synthesis system.	[57]
Robotic nanofabrication	Learns scanning-probe manipulation under uncertainty and sparse feedback.	Provides a control analogue for adaptive surface or interface modification.	Manipulates individual molecules on a model surface rather than a cathode interface.	[58]
RL-based retrosynthesis reasoning	Trains RetroDFM-R using chemically verifiable rewards for retrosynthetic reasoning.	Relevant to route planning for organic cathodes and coating precursors.	Primarily evaluated on organic-retrosynthesis benchmarks.	[59]
End-to-end retrosynthesis planning	Refines coherent, long-horizon synthesis-route generation using RL.	Provides a future analogue for multistep organic-cathode synthesis planning.	No experimental or cathode validation provided.	[60]

**Table 5:** Reinforcement -learning algorithm families relevant to cathode-material design.

RL Family or Formulation	Suitable Cathode-Design Task	Typical Action Space	Quantitative Selection Guide	Primary Limitation	Representative Evidence	Ref.
Value-based RL/DQN	Discrete composition, dopant, and defect editing	Add, remove, or substitute elements; select dopants; create vacancies.	Finite discrete actions and short trajectories; validity and success at a fixed episode budget. MolDQN used 20–40 edits and achieved 100% validity.	Poor scalability for very large or continuous action spaces.	MolDQN: valid molecular edits with multi-objective rewards.	[39]
Policy-gradient RL	Generative construction of cathode-related candidates	Generate SMILES tokens, graph components, fragments, or compositional units.	Variable-length stochastic generation; comparison of validity and target-hit rates. REINVENT obtained approximately 95% validity and 98% target satisfaction.	Reward hacking, mode collapse, and invalid candidates.	REINVENT: reward-guided molecular generation.	[35]
Actor–critic RL	Large or continuous chemical and geometric searches	Propose continuous chemical or geometric moves and estimate their value.	Continuous or high-dimensional actions; geometric and energy errors. Reported transition-state RMSDs were below 0.1 Å.	Sensitive to critic error, reward scaling, and unstable updates.	Molecular traversal and minimum-energy-path optimisation.	[61]
Model-based and offline RL	Synthesis and processing optimisation from historical or simulated data	Adjust temperature, concentration, atmosphere, or processing conditions over time.	Use when direct interaction is expensive and offline data are available; report dataset size and surrogate error. 10,000 simulations with phase-fraction error below 0.1.	Model bias under distribution shift.	Offline RL for MoS_2_ CVD synthesis schedules.	[53]
Goal-conditioned RL	Design toward variable cathode-property profiles	Condition candidate edits on a target-property vector.	Use when one policy must satisfy multiple target values; set success tolerance from predictor uncertainty. Tolerance: ±0.15 eV with 100% structural validity.	Requires reachable goals and calibrated predictors.	Inverse design of organic semiconductor molecules.	[62]
Knowledge-augmented RL	Data-scarce composition and processing design	Use expert rules, trusted experience, and model-refinement feedback.	Sparse samples relative to design dimensionality; assess guidance-model accuracy and joint success rate. AIMATDESIGN reported AUC = 0.95 and joint success = 50.32%.	Expert knowledge may overconstrain exploration.	AIMATDESIGN: knowledge-based rewards and model refinement.	[63]
Crystal-structure RL	Crystal search, structural relaxation, and site ordering	Swap sites, move atoms, or alter local ordering.	Energy feedback was available after each structural move; compare steps or time to the global minimum. RL used 71% of the uniform-policy steps and saved up to 46% computation time.	Low structural energy does not ensure electrochemical performance.	Adaptive RL for crystal-structure prediction.	[44]
Multi-objective RL	Balance voltage, capacity, stability, cost, and synthesizability	Evaluate edits against several competing objectives.	Two or more conflicting objectives; objective-wise and joint success or Pareto coverage. 100,000 quantum-chemistry calculations and 250 MCTS samples per rollout.	Scalarisation can conceal trade-offs.	AlphaZero optimisation of redox potential, stability, and synthesizability.	[37]
Active-learning-coupled RL	Search with expensive DFT or experimental rewards	Select informative candidates for oracle evaluation and update the policy.	Use when oracle cost dominates; compare hits per oracle call and time per hit. RL–AL produced 5–66-fold more hits and 4–64-fold lower computational time.	Surrogate error and acquisition bias may distort learning.	Improved oracle-call efficiency in molecular design.	[43]

## Data Availability

Data can be provided upon request.
